# Visualizing *Escherichia coli* Sub-Cellular Structure Using Sparse Deconvolution Spatial Light Interference Tomography

**DOI:** 10.1371/journal.pone.0039816

**Published:** 2012-06-28

**Authors:** Mustafa Mir, S. Derin Babacan, Michael Bednarz, Minh N. Do, Ido Golding, Gabriel Popescu

**Affiliations:** 1 Department of Electrical and Computer Engineering, University of Illinois at Urbana-Champaign, Urbana, Illinois, United States of America; 2 Beckman Institute for Advanced Science & Technology, University of Illinois at Urbana-Champaign, Urbana, Illinois, United States of America; 3 Department of Physics, Center for the Physics of Living Cells, University of Illinois at Urbana-Champaign, Urbana, Illinois, United States of America; 4 Verna and Marrs McLean Department of Biochemistry and Molecular Biology, Baylor College of Medicine, Houston, Texas, United States of America; Friedrich-Schiller-University Jena, Germany

## Abstract

Studying the 3D sub-cellular structure of living cells is essential to our understanding of biological function. However, tomographic imaging of live cells is challenging mainly because they are transparent, i.e., weakly scattering structures. Therefore, this type of imaging has been implemented largely using fluorescence techniques. While confocal fluorescence imaging is a common approach to achieve sectioning, it requires fluorescence probes that are often harmful to the living specimen. On the other hand, by using the intrinsic contrast of the structures it is possible to study living cells in a non-invasive manner. One method that provides high-resolution quantitative information about nanoscale structures is a broadband interferometric technique known as Spatial Light Interference Microscopy (SLIM). In addition to rendering *quantitative phase* information, when combined with a high numerical aperture objective, SLIM also provides excellent depth sectioning capabilities. However, like in all linear optical systems, SLIM's resolution is limited by diffraction. Here we present a novel 3D field deconvolution algorithm that exploits the sparsity of phase images and renders images with resolution beyond the diffraction limit. We employ this label-free method, called deconvolution Spatial Light Interference Tomography (dSLIT), to visualize coiled sub-cellular structures in *E. coli* cells which are most likely the cytoskeletal MreB protein and the division site regulating MinCDE proteins. Previously these structures have only been observed using specialized strains and plasmids and fluorescence techniques. Our results indicate that dSLIT can be employed to study such structures in a practical and non-invasive manner.

## Introduction

The field of cell biology emerged in the 17^th^ century, when van Leeuwenhoek used a light microscope to observe microscopic objects including bacteria and human cells. However, since its inception, cell microscopy has contended with two major issues: lack of contrast, due to the thin and optically transparent nature of cells, and diffraction limited resolution. The hard limit on diffraction limited resolution was first calculated by Abbe in 1873 [1] to be approximately half the wavelength of the illumination light. In order to improve contrast, the approach is either to engineer *exogenous* contrast agents or to exploit the optics of light-specimen interaction and reveal the *endogenous* contrast provided by naturally occurring structures [2]. Currently, fluorescence microscopy is the most commonly used technique in cell biology because it provides very high (theoretically infinite) contrast and also allows for labeling specific structures [3]. The key development that essentially combined the intrinsic and exogenous contrast imaging fields is to genetically engineer the cell to express green fluorescent protein (GFP) [4]. The advent of this technology made it possible to genetically modify a cell such that it naturally expresses GFP and binds it to prescribed cellular structures, allowing the imaging of living cells.

Over the past two decades, fluorescence microscopy has also enabled a number of super-resolution technologies, including Stimulated Emission Depletion microscopy (STED) [5], Stochastic Optical Reconstruction Microscopy (STORM) [6], (Fluorescence) Photo-Activated Localization Microscopy, (f)PALM [7], [8], Spatially Structured Illumination Microscopy (SSIM) [9], etc., collectively referred to as *far-field nanoscopy* techniques (for a review, see Ref. [10]). Impressively, these methods provide a transverse resolution of 20–30 nm. Abbe's resolution limit is overcome by taking advantage of the *nonlinear* properties (eg. saturation, switching) of the engineered fluorophores. Since these methods require scanning or many frames to reconstruct the final image, they are often limited by severe tradeoffs between acquisition time and field of view. More importantly, the nonlinear light-specimen interactions require a high level of illumination intensity, which in turn adds limitations due to photodamage and photobleaching.

For improving *endogenous* contrast imaging there are two widely used methods, namely, Differential Interference Contrast (DIC or Nomarski) and Phase Contrast [3]. Both of these techniques rely on the realization by Abbe that image formation, and thus contrast generation, is due to interference between scattered and unscattered waves [1]. It was this concept that allowed Zernike to develop phase contrast microscopy [11] which improves the contrast of the image by introducing a quarter wavelength shift between the light scattered by the specimen and the un-scattered light. Phase contrast opened many new doors in live cell imaging; however the information from a phase contrast image is qualitative (intensity is not linearly proportional to phase) and, of course, the resolution is still diffraction limited.

Since it has become increasingly clear that to truly understand cellular function, it is necessary to image with high resolution in three dimensions, many of the fluorescence techniques mentioned above have also been adapted to work in 3D. Confocal microscopy is the most commonly used technique for 3D imaging and provides an axial resolution of approximately 500 nm [12]. 4Pi microscopy yields an axial resolution of 90 nm [13], while, more recently, 3D-Storm provides 50 – 60 nm resolution [14]. Another approach for 3D re-construction is deconvolution microscopy, in which the blurring of the fluorescence image due to diffraction is treated as a linear problem and reversed numerically. Note that, of course, only the amplitude (intensity) of the field is measured in all these methods.

Here we show that if instead of just measuring intensity, the complex field (i.e., phase and amplitude) is measured, the 3D reconstruction of the specimen structure can be obtained without the need for exogenous contrast agents. Measuring the phase shift that the specimen adds to the optical field at each point in the field of view is known as *quantitative phase imaging* (QPI) [2]. This field has been developing rapidly over the past decade and recently a variety of methods have been developed [2],[15],[16],[17],[18],[19]. These advances in QPI methods have enabled three dimensional optical tomography of transparent biological samples using Radon transform based algorithms that were originally developed for X-ray computed imaging [20], [21], [22]. QPI based projection tomography has also been demonstrated on live cells with several approaches demonstrating high resolution [23], [24], [25], [26], [27], [28].

Recently, we have developed a new QPI modality known as Spatial Light Interference Microscopy (SLIM) [29]. SLIM is a broadband (white light) illumination technique that provides phase sensitive measurements of thin transparent structures with unprecedented sensitivity [30], [31]. By combining the short-coherence length of the broadband illumination with a high numerical aperture objective, SLIM provides depth sectioning capabilities [32]. Combining 3D SLIM images with a linear forward model based on the first order Born approximation, it has recently been shown that it is possible to perform label-free optical tomography in a technique referred to as Spatial Light Interference Tomography (SLIT) [32]. SLIT operates by measuring the 2D complex field while translating the focus position in increments of less than half the depth of field. The measured 3D complex field is then deconvolved using an *experimentally* measured three-dimensional point spread function (PSF). This tomographic capability has been demonstrated successfully on live neurons and photonic crystal structures [32].

Despite the advantages provided by SLIM, its resolution is still diffraction limited [33]. Such degradations are common to all optical instruments and may be reduced to an extent through post-processing methods such as deconvolution. Deconvolution works by inverting the optical transfer function of the instrument and has been widely used in intensity based techniques [34], [35]. However, such methods have not been investigated thoroughly on complex fields measured by QPI instruments. Previous work [36] suggests that the noise-amplification that is commonly encountered when applying deconvolution to intensity images is not significant when they are applied to complex field measurements. The high SNR phase measurements obtained by SLIM provide a far more accurate modeling of the convolution with the PSF of the optical system in the complex fields, compared to the approximate convolution model typically used for intensity based methods. So far two novel deconvolution methods have been developed for SLIM. First, a non-linear method [37] was developed that estimates the unknown amplitude and phase through a combination of variable projection and quadratic regularization on the phase. The second method, called dSLIM [38], is based on modeling the image using sparsity principles. This type of modeling is very effective in capturing the fine-scale structural information that is lost due to the instruments optical transfer function. It was shown that dSLIM provides a resolution increase by a factor of 2.3, enabling super-resolution imaging with SLIM. Here we present *sparse deconvolution spatial light interference tomography (dSLIT)*. This new method provides super-resolution in 3D and allows us to study the fine scale sub-cellular structure present in *E. coli*.

The idea that the sub-cellular environment of *E. coli* cells is simply an amorphous mix has been proven to be incorrect, mostly due to the availability of high-resolution fluorescence methods. Numerous structures and distinct localizations of proteins have been studied such as the MinCD complex [39], [40], FtsZ [41], [42], [43] and MreB [40]. Furthermore, localization and structure has also been observed in the deposition of Lipopolysaccharides [44]. Interestingly many of these proteins have been found to lie in a helical or coil formation. The nature of these helices and coils is still under active investigation and many important questions remain to be answered. However, studying these sub-cellular structures using fluorescence requires specialized strains and probes, which inhibit the observation of these structures in wild-type strains in a non-invasive manner. In this work, we show that dSLIT can be used to render high-resolution images of the three-dimensional subcellular structure in *E. coli* cells. We find that dSLIT can characterize the behavior and interactions of these structures without using fluorescent labels.

## Methods

### *E. coli* culture and imaging

*E. coli* MG1655 cells are cultured overnight in LB (Luria Broth) and then sub-cultured by 100x dilution into commercial M9CA media with Thiamine (Teknova M8010) until they reach an optical density (OD) of ∼0.2. The cells are then concentrated to an OD of ∼0.4 and 2 µl of the culture is pipetted onto a glass bottom dish (In Vitro Scientific D29-21-1-N) and covered by a 1 mm thick agar slab (1.5% Agarose, M9CA media). In order to mitigate drying of the agar, 70 µl of H_2_O is carefully pipetted onto the edge of the dish, ensuring that it never makes contact with the sample. The dish is then covered with a circular coverslip to reduce the effects of evaporation. During imaging the cells are kept at 37°C by an incubator (XL S1 w/ CO2 kit, Zeiss) fitted on the microscope (AxioObserver Z1, Zeiss). Images are acquired using 63x/1.4 Oil phase contrast objective (Zeiss). For each field of view the sample is scanned in z with a slice spacing of 0.280 µm with a total of 15 slices. The exposure time for each image is 35 ms with the lamp temperature at 3200K. The images are then processed to retrieve the quantitative phase maps and then de-convolved as described below.

### Spatial Light Interference Microscopy

**Figure 1:**
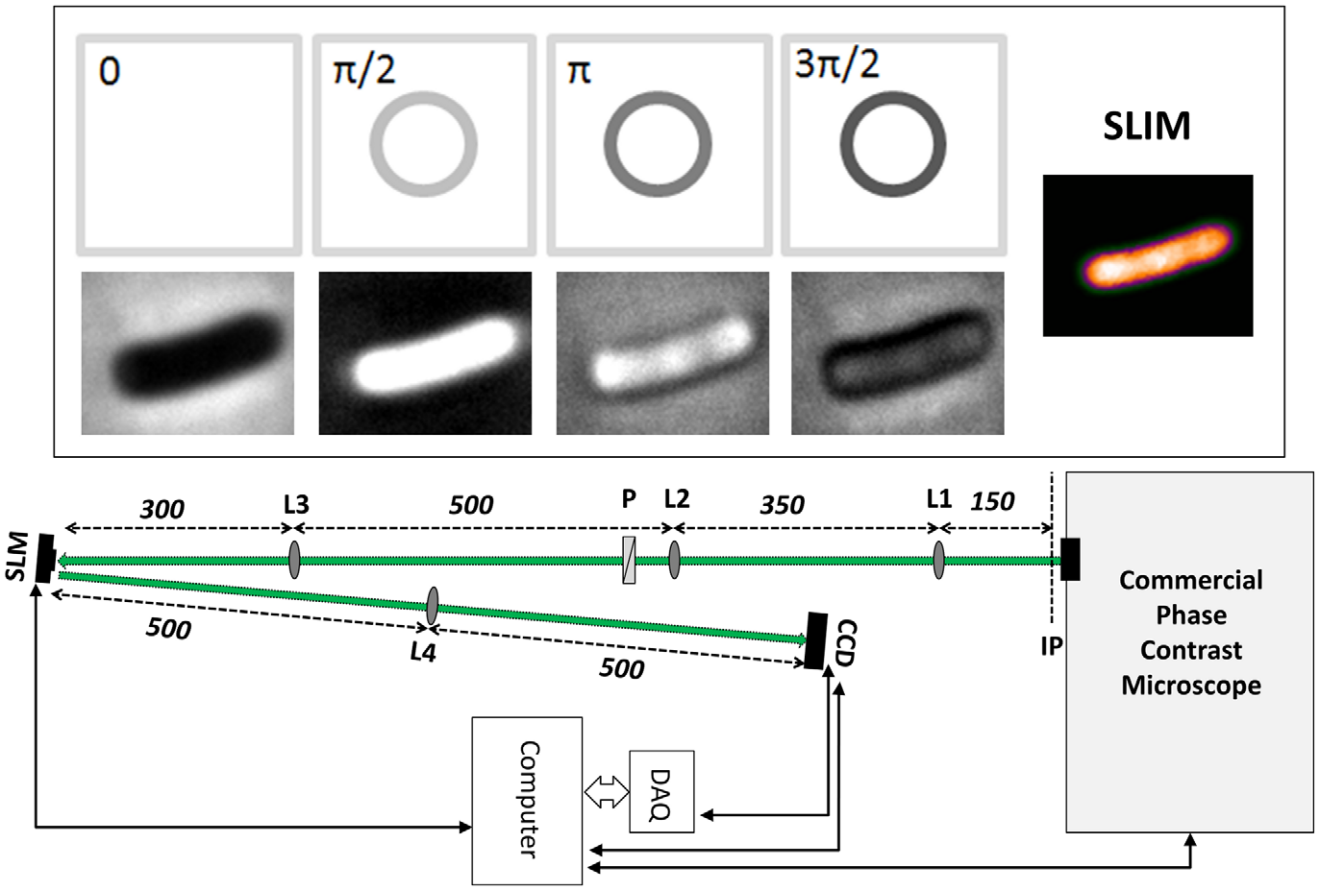
Experimental setup. The SLIM module is attached to a commercial phase contrast microscope (AxioObserver Z1, Zeiss). The first 4-f system (lenses L1 and L2) expands the field of view to maintain the resolution of the microscope. The polarizer, P is used to align the polarization of the field with the slow axis of the Spatial Light Modulator (SLM). Lens L3 projects the back focal plane of the objective, containing the phase ring, onto the SLM which is used to impart phase shifts of 0, π/2, π and 3π/2 to the un-scattered light relative to the scattered light as shown in the inset. Lens L4 then projects the image plane onto the CCD for measurement. From the 4 intensity measurements a quantitative phase map is reconstructed as shown in the inset.

SLIM is a recently developed broadband QPI technique, which combines the high contrast intensity images acquired by phase contrast microscopy with quantitative information acquired through holography [29], [45]. This combination allows for imaging using the intrinsic contrast of the sample and provides quantitative optical path length information at each point in the image. A schematic for the SLIM experimental setup is shown in Fig. 1. SLIM is designed as an add-on module to a commercial phase contrast microscope (AxioObserver Z1 in this case). In conventional phase contrast microscopy, a thin metal annulus (phase ring) located at the back focal plane of the objective lens is used to introduce a π/2 phase shift between the light scattered by the sample and the un-scattered light, thus coupling the phase information into the intensity map that is observed by eye or measured by a CCD. Although phase contrast revolutionized optical microscopy and is a ubiquitous tool in cell biology, it does not provide any quantitative information about the sample. In SLIM the back focal plane of the objective is projected onto a programmable liquid crystal spatial light modulator (SLM, Boulder Nonlinear). The pattern on the SLM is modulated to precisely match the phase ring of the objective and is then used to impart a controllable phase shift between the scattered and un-scattered light. By recording four intensity maps at phase shifts of 0, π/2, π and 3π/2 (Fig. 1 inset), it is possible to uniquely determine the actual phase shift imparted by the sample relative to its surroundings. This phase shift is linearly proportional to the refractive index and the thickness of the sample. References [29], [45] present more details on the experimental setup. Prior to any further processing or analysis the images are unwrapped to correct any 2π phase ambiguities using Goldstein's algorithm. This is usually unnecessary for optically thin samples such as living cells since the measured phase is consistently below 2π.

Due to the broadband (short coherence length of 1.2 µm) of the illumination source SLIM does not suffer from reduced resolution due to speckle which has plagued previous QPI methods and, due to the common path geometry, it is extremely temporally sensitive. SLIM's spatial and temporal sensitivity to optical path length have been measured to be 0.28 nm and 0.029 nm respectively. Another unique feature of SLIM as a QPI technology is that it can be overlaid with any other microscopy modality (e.g., epi-fluorescence) that is available commercially without any additional effort. These capabilities have enabled applications ranging from nanoscale topography and refractometry [31] to quantifying intracellular transport [46], [47], blood screening [48], cancer detection [49] and cell growth [30], [50].

When the short coherence length of the illumination is coupled with the shallow depth of field provided by a high numerical aperture objective, SLIM provides excellent depth sectioning capabilities. In this work we combine the depth sectioning capabilities of SLIM with a new 3D sparse deconvolution method that allows for sub-diffraction limited resolution. Using this method we are able to resolve sub-cellular structures in *E. coli* which are invisible in SLIM. The deconvolution method is described in detail below.

### 3D Complex Field Deconvolution via Sparsity

The following notation is used in this section: Bold letters **h** and **H** denote vectors and matrices, respectively, with transposes **h** ^T^ and **H** ^T^. The spatial coordinates within an image are denoted by (*x*, *y*, *z*), operator * denotes convolution, and *i* is equal to √−1. Finally, {·} is used to denote a set created with its argument.

As described in reference [32], under the first order Born approximation, the 3D complex field distribution measured by SLIM, *U*(**r**)  =  |*U*(**r**)|exp[*i*Φ(**r**)], can be expressed as a convolution between the susceptibility of the object, 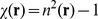 (where *n* is the refractive index) and the point spread function of the microscope, *h*(**r**). Essentially, the imaging system acts as a band pass filter in the spatial frequency domain. Thus the measured field can be written as 
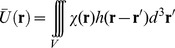
, where 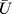 is the complex analytic signal associated with the real field. The PSF, h, can be determined within the Born approximation by considering the contribution of all the optical components in the system. However, this calculation only provides the response of an idealized system. Thus, we measured the PSF experimentally by imaging microspheres with diameters less than the size of the diffraction spot such that they essentially behave as point scatterers. Therefore, for the purposes of the deconvolution procedure presented here we may model the measured field, 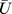, as
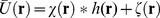
(1)where 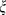
(**r**) is the additive signal independent noise. Generally, both the magnitude and the phase of the image function are affected by the PSF and the noise. However, the degradation in the magnitude field is much smaller compared to the degradation in the phase [36]. Moreover, most of the useful biological information is contained in the phase image, and the magnitude image is not of much interest. Therefore, it is reasonable to assume that the magnitude of the image is constant and passes through the instrument without degradation, i.e., 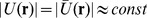. This assumption allows us to remove the magnitude component, and write the deconvolution problem solely in terms of the phase Φ (**r**) as
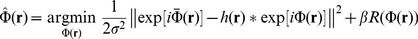
(2)where σ2 is the noise variance, and *R*(·) is the regularization functional used to enforce certain image properties during deconvolution. Let us now denote by *g*(**r**) the field 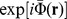 acquired by the microscope, and by *f*(**r**) the unknown field 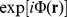 we are trying to recover. These fields can be represented as vectors **g** and **f**, respectively, by stacking the images as single columns with N pixels. Using this representation, the image formation in (1) can be written in matrix-vector form as
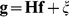
(3)where **H** is the convolution matrix corresponding to the PSF *h*(**r**). Similarly, the deconvolution problem (2) can be expressed as



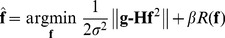
(4)The formulation in (4) can be expressed within the Bayesian framework by defining an observation model p(**g**|**f**, *σ* ^2^) and an image prior p(**f**) as follows



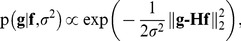
(5)

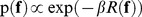
(6)


The optimization problem in (4) then corresponds to finding the maximum (the mode) of the joint distribution p( **g**|**f**, *σ* ^2^)  =  p( **g**|**f**, *σ* ^2^) p(**f**), corresponding to a maximum *a posteriori* (MAP) estimation. In the following, we will follow the Bayesian modeling formulation.

Notice that in (5), the signal-independent noise **n** is modeled as zero-mean, independent white Gaussian noise with variance σ^2^. The Gaussian modeling accurately describes the noise characteristics in SLIM, since the signal-to-noise ratio (SNR) is very high (as in fluorescence microscopy [34]). In addition, the noise variance σ^2^ can be estimated experimentally from a uniform area in the acquired image.

As mentioned earlier, the functional *R*(·) (and thus the image prior p(**f**)) is used to regularize the solution by enforcing certain image characteristics. The inverse filter solution can be obtained when no regularization is used (R = 0), but this approach generally does not produce good results due to excessive amplification of noise. The role of regularization is to impose desired characteristics on the image estimates, and to suppress the noise and ringing artifacts. The regularization parameter β controls the trade-off between the data-fidelity and the strength of the regularization on the estimates.

We now present an image model suitable for characterizing both the specimen and the image instrument. Phase contrast imaging provides high sensitivity at the sharp object boundaries, but it is relatively insensitive to slow-variations in the background region. Thus, phase images generally exhibit high contrast around edges corresponding to e.g., cell boundaries, which in turn provides accurate morphological information. In addition, in live cell imaging, the specimen contains a fine structure and small-scale dynamics.

Based on these characteristics, we propose to use the sparse representation/reconstruction framework [51] that is suitable for modeling phase images. Sparse representation and reconstruction has recently been used in a number of imaging problems with great success (see, e.g., [52], [53]. It has also been shown [52], [54] that sparsity-based deconvolution is generally superior compared to classical methods based on Wiener filtering and Tikhonov regularization.

As demonstrated below, phase images can be accurately represented sparsely in some transform domain, that is, when an appropriate transform is applied to the images, most of the transform coefficients become very small while only a few contain most of the signal energy. This transform sparsity allows us to capture the characteristics of spatial variations within the image. In this work, we use a collection of *L* linear transforms **D**_k_ with *k = 1, ..., L*, which are chosen as difference operators that capture spatial variation at varying scales and orientations. Specifically, we use the first and second order directional difference operators



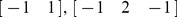
(5)


and 45° and −45° first-order derivative filters



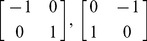
(6)


**Figure 2:**
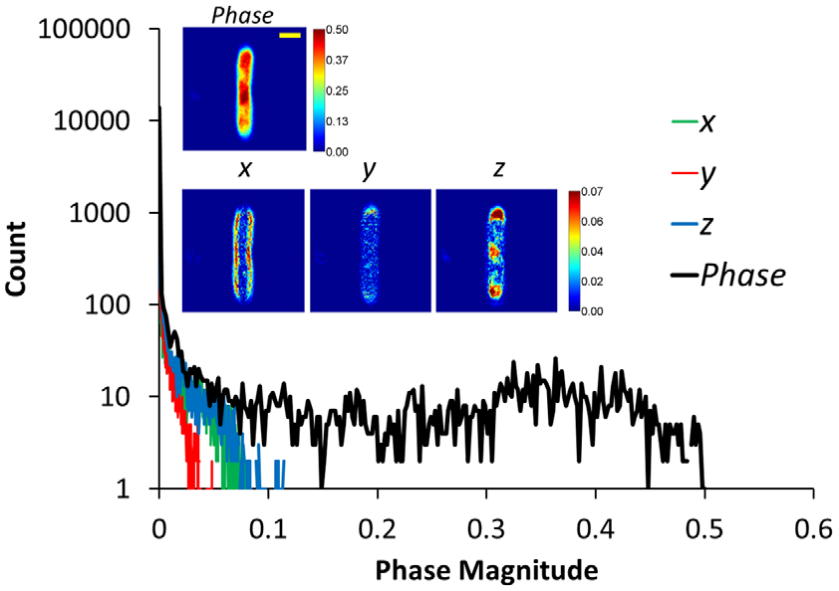
Sparsity property of phase images. Images show the original phase image, and the output images obtained by applying first order directional derivatives in the x, y, and z directions, as labeled, scale bar is 1 µm. The plot shows the corresponding log-histograms, the increase in sparsity is clearly visible.

These 2D transforms are applied on all three planes in the image, that is, on *x−y, y−z and x−z* planes, which in total give 12 transforms to capture the local spatial variations within the 3D structure. More complicated transforms can also be incorporated in the proposed framework in a straightforward manner. As an illustration of the sparsity property of phase images, a SLIM phase image and the output of applying difference operators (in x-, y- and z- directions) are shown in Fig. 2, along with the corresponding log-histograms. It is evident that most of the structural information is accurately captured by the filtered images. In addition, the cell structure concealed in the acquired phase image is revealed in the filtered images (especially in the z-direction). Notice also that compared to the SLIM image, the sparsity level is significantly increased and the decrease in resolution due to the PSF can clearly be observed in the filtered images.

Using these transforms, the image model can be constructed to exploit the sparsity in the transform coefficients. In this work, we employ separate Gaussian priors on each transform coefficient as



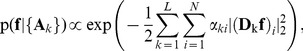
(7)where 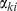 are the weighting coefficients. The prior in (7) can be expressed in a more compact matrix-vector form as



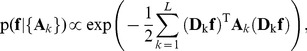
(8)where **A** *_k_* are diagonal matrices with 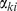,*i =  1, . . .N* in the diagonal. The prior in (7) constitutes a sparse image prior, since the transform coefficients (**D** *_k_* **f**)*_i_* at pixel *i* are suppressed when the corresponding weight 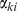 assumes very large values.

The weights 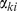 also represent the local spatial activity at each location, and hence they are a measure of spatial variation in the corresponding filters direction. Since we do not know a *priori* which transform coefficients should be suppressed, they are estimated simultaneously with the image. For their estimation, we assign uniform priors as 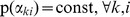
(9)


It should be noted that this image modeling based on the sparsity principle is used solely as an image prior, and it does not necessarily result in image estimates that are sparse in the transform domains. This is because the image estimate is still constrained with the acquired image **g** via the data constraints (the first term in (4)). Real images are generally only approximately sparse, with a few transform coefficients containing the majority of the image energy while the remaining majority of the coefficients have very small values. These small values may carry information about the subtle image features. The modeling employed in this work allows for adaptively estimating both the large and small coefficients by estimating the parameters 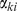 simultaneously with the image.

Using the models for the noise in (5), the image in (8), and the parameters in (9), the problem of estimating the unknown complex image f and the weights 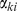 is formulated within the MAP framework as



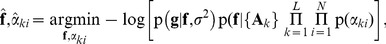
(11)




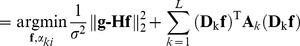
(12)


We solve this problem using an alternating iterative minimization scheme where each unknown is estimated by keeping other variables fixed. Notice that this problem is convex in f and *α_ki_*, but it is not jointly convex. For such problems, alternating minimization is shown to be an effective strategy, and it converges to a local minimum of the objective function (see 55,56] for related discussions).

The estimate of the complex image **f** is found by taking the derivative of (12) and setting it equal to zero, which yields



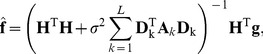
(13)


The parameters 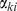 are estimated in a similar fashion by minimizing (12), which gives the update



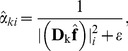
(14)where *ε* is a small number (e.g., 10^−6^) used to avoid the trivial solution (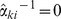) for numerical stability. It is evident from (14) that the parameters 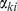 are functions of the k^th^ filter response at pixel i of the image estimate 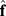. Thus, the strength of the enforced sparsity is varied spatially within the image, and it is adaptively estimated with each new image estimate. Through the use of the transforms, this can also be seen as controlling the amount of spatially-varying smoothness applied on the image estimate: When a parameter 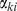 assumes a large value, a higher amount of smoothness is applied at pixel *i* (and vice versa). Low values of 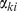 will therefore be obtained in the areas with more edge structure, preserving the image features.

In summary, the dSLIT deconvolution method estimates the complex image **f** using (13) and the parameters 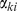 using (14) in an alternating fashion. Estimation of the image in (13) is performed using the conjugate gradient (CG) method. The operations involving the products with matrices **H** and **D** *_k_* are done via multiplications in the Fourier domain. As mentioned earlier, the noise variance σ^2^ is estimated from an approximately uniform background region in the image.

The alternating minimization method employed here can be shown to belong to the family of half-quadratic minimization methods, for which certain theoretical convergence guarantees exist [55], [56]. The method is initialized with the acquired phase image **g** without any pre-processing. Since the noise level in SLIM is very low, this image is a good estimate of the sharp image. In addition, the experimentally obtained point spread function (PSF) **h** accurately represents the true PSF (see next section). Empirically we found that the proposed deconvolution algorithm is very robust and generally converges very rapidly within a few iterations. Finally, note that since the deconvolution is applied directly in the complex image domain, dSLIT does not alter the quantitative imaging property of SLIM. In contrast, traditional deconvolution methods applied on intensity images cannot preserve the quantitative information.

## Results

### Deconvolution Results

**Figure 3:**
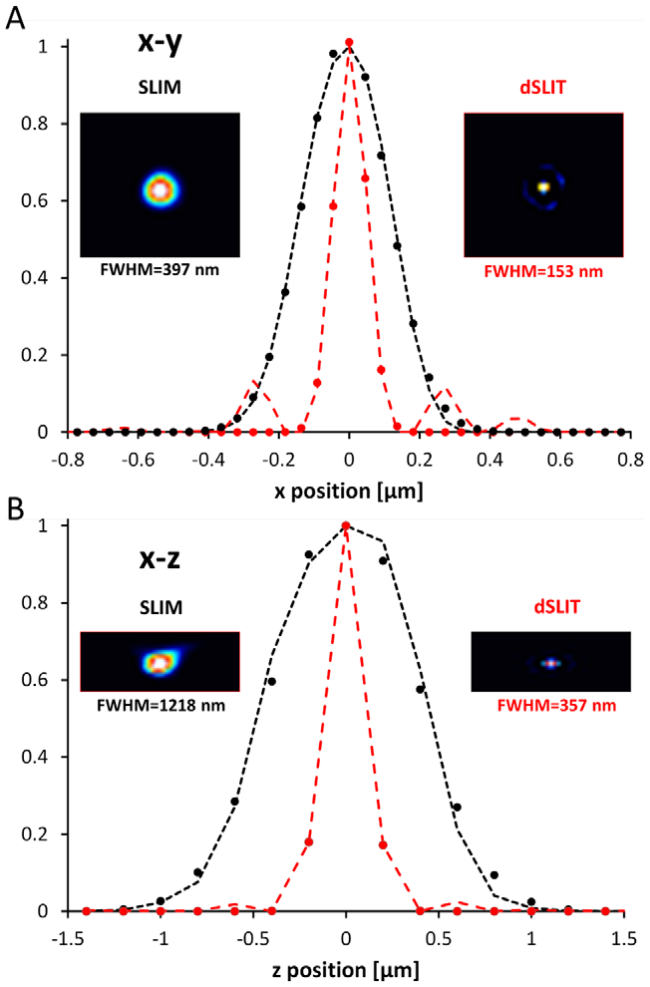
Three dimensional point spread function. A) Comparison of raw and deconvolved. PSF in the x-y plane; the deconvolution process reduces the FWHM from 397 nm to 153 nm. B) Comparison of raw and deconvolved PSF in the x–z plane; the deconvolution process reduces the FWHM from 1218 nm to 357 nm. The dashed lines show the data and the circular markers indicate the Gaussian fit used to determine the FWHM.

To quantify the increase in resolution we applied dSLIT to the experimentally measured point spread function (PSF). The experimental PSF was acquired by using SLIM to measure a sub-resolution (150 nm) diameter polystyrene bead, while scanning the focus in z in increments of 200 nm. Fig. 3 shows the results of the deconvolution, the FWHM were calculated by fitting the experimental results with a Gaussian. In the x–y plane (Fig. 3A) an increase in resolution of 2.5 times is achieved as the FWHM is decreased from 397 nm to 153 nm. In the axial (z) direction the FWHM is reduced from 1218 nm to 357 nm, corresponding to an increase in resolution of 3.4 times that of SLIM (Fig. 3B).

### 3D Subcellular Structures in *E. coli*

**Figure 4:**
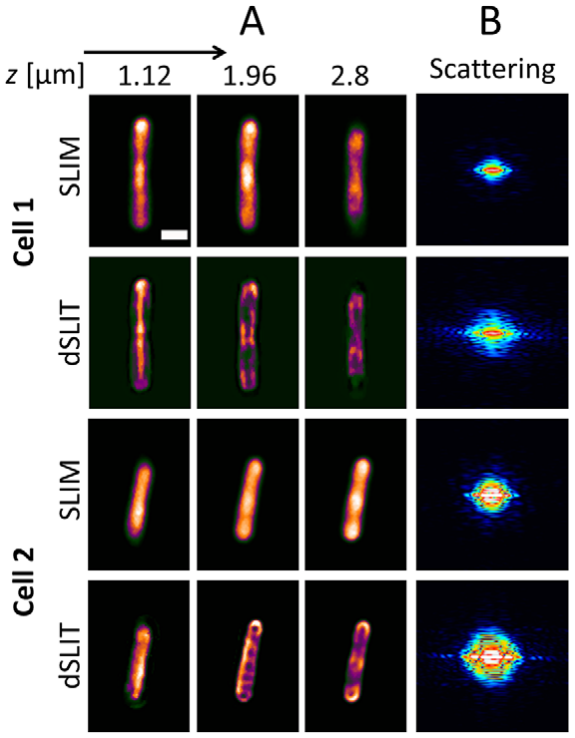
Comparison of raw and deconvolved data from two cells. A) SLIM and dSLIT images at a variety of z-positions, with clearly visible coiled structures. B) Scattering maps corresponding to the images shown in A. The increase in resolution is clearly visible from the extra information at higher angles in the dSLIT maps.

*E. coli* cells were prepared and imaged as described above. Fig. 4A shows a comparison of the SLIM and dSLIT images from two cells at different z- positions. It is clear that the deconvolution process reveals subcellular structure that is not visible in the SLIM images. Fig. 4B shows the center slice of the 3D Fourier transform of the SLIM and dSLIT data. As discussed above SLIM measures the complex field at each z-position thus the intensity distribution in far field or the scattering plane may be determined by calculating its Fourier Transform, a technique known as Fourier Transform Light Scattering (FTLS) 57]. Comparing the scattering maps obtained from the SLIM and deconvolved data, it is clear that there is more information available at higher scattering angles or spatial frequencies corresponding to smaller structures. Thus combining dSLIT with FTLS provides scattering information at the sub-cellular level for single *E. coli*. To our knowledge the scattering from single *E. coli* and their sub-cellular structures has not been studied, probably due to the practical difficulties involved in performing such measurements.

**Figure 5:**
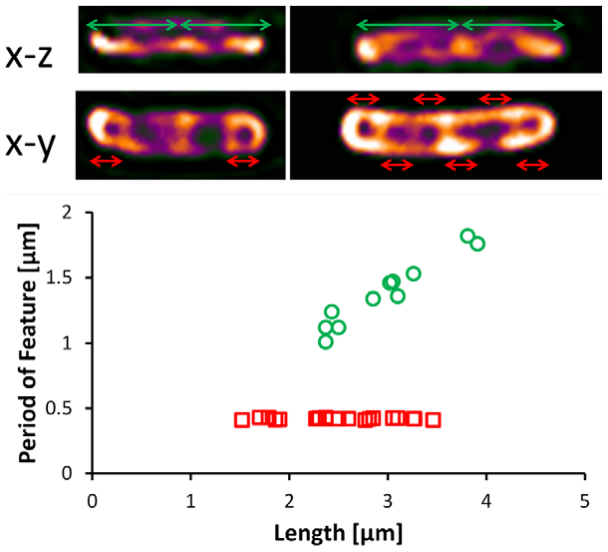
Measurement of prominent structures found in 26 cells. In the x–y plane a coil structure is visible that has a period of approximately 0.43 µm and does not vary with the length of the cell. In the x-z plane another structure is visible that has a period of half the cell-length.

The dSLIT data reveal two sets of subcellular coil-like structures that are visible in most of the cells that were analyzed; Fig. 5 summarizes the measurements made on these structures. In the x–y plane a coiled structure is observed with an average period of 430 nm. Although the clarity and completeness of the structure varies from cell to cell, the period of the structure was measured to be invariant with the length of the cell. In the x–z plane another coil-like structure is apparent, which was measured to have a period of approximately half the length of the cell. This structure is not readily visible in smaller or freshly divided cells. The differences observed in x-y and x–z plane are likely due to the difference in resolution of the method in the axial and lateral planes. Such coil like structures have been observed in several contexts in *E. coli* cells including the MreB cytoskeletal element, MinCDE coiled arrays, outer membrane proteins and lipopolysaccharide [40], [44]. Fluorescence measurements of these structures indicate that they are most likely functionally distinct though little is known about their temporal behavior. Although dSLIT reveals these structures, there is no way to truly determine from the current data what the structures truly are. For this, it is necessary to conduct a study in which different subcellular structures are fluorescently labeled. Once the identity of the structure is determined it will then be possible to study it in a label free manner using dSLIT. This will enable practical experiments of the behavioral dynamics of these sub-cellular structures without the need for specialized strains or probes.

## Discussion

In this paper we presented dSLIT, a novel deconvolution microscopy method that retrieves sub-diffraction limited resolution information from the complex fields measured by SLIM. dSLIT operates on three key observations. First, the degradation of the image by a microscopy PSF can be modeled as a linear process. Second, due to the high SNR characteristic of SLIM, this PSF may be measured experimentally. Third, the quantitative phase measurements of thin biological specimen, like *E. coli* cells, can be accurately modeled using sparsity principles. These properties of the measurement system allow for a very effective deconvolution process with a 2.5x resolution increase in the longitudinal resolution and a 3.4x increase in axial resolution, as shown in Fig. 3. This increase in resolution allowed us to measure sub-cellular structure in *E. coli* that was previously not visible in the SLIM data. Using dSLIT we found two consistent coil-like subcellular structures in *E. coli*, one that retains a constant period as the cell grows and one with a period of approximately half the length of the cell. Although several such structures have been previously identified, little is known about their function and behavior due to the practical difficulties involved in imaging them. The results presented here indicate that dSLIT can be used to characterize and study such sub-cellular structure in a practical and non-invasive manner, opening the door for a more in depth understanding of the biology.

## References

[1] Abbe E (1873). Beitrage zur Theorie des Mikroscps und der mikrskopischen Wahrnehmung.. Arch Mikrosk Anat 9.

[2] Popescu G (2011). Quantitative phase imaging of cells and tissues. New York: McGraw-Hill.. 362 p.

[3] Stephens DJ, Allan VJ (2003). Light microscopy techniques for live cell imaging.. Science.

[4] Tsien RY (1998). The green fluorescent protein.. Annual Review of Biochemistry.

[5] Hell SW, Wichmann J (1994). Breaking the Diffraction Resolution Limit by Stimulated-Emission – Stimulated-Emission-Depletion Fluorescence Microscopy.. Optics Letters.

[6] Rust MJ, Bates M, Zhuang XW (2006). Sub-diffraction-limit imaging by stochastic optical reconstruction microscopy (STORM).. Nature Methods.

[7] Betzig E, Patterson GH, Sougrat R, Lindwasser OW, Olenych S (2006). Imaging intracellular fluorescent proteins at nanometer resolution.. Science.

[8] Hess ST, Girirajan TPK, Mason MD (2007). Nanoscale visualization of fluorescent protein distributions in cells by fluorescence photoactivation localization microscopy (FPALM).. Biophysical Journal.

[9] Gustafsson MGL (2005). Nonlinear structured-illumination microscopy: Wide-field fluorescence imaging with theoretically unlimited resolution.. Proceedings of the National Academy of Sciences of the United States of America.

[10] Hell SW (2009). Microscopy and its focal switch.. Nat Methods.

[11] Zernike F (1955). How I Discovered Phase Contrast.. Science.

[12] Wright SJ, Wright DJ (2002). Introduction to confocal microscopy.. Cell Biological Applications of Confocal Microscopy, Second Edition 70: 1−+.

[13] Hell S, Stelzer EHK (1992). Fundamental Improvement of Resolution with a 4pi-Confocal Fluorescence Microscope Using 2-Photon Excitation.. Optics Communications.

[14] Huang B, Wang WQ, Bates M, Zhuang XW (2008). Three-dimensional super-resolution imaging by stochastic optical reconstruction microscopy.. Science.

[15] Ikeda T, Popescu G, Dasari RR, Feld MS (2005). Hilbert phase microscopy for investigating fast dynamics in transparent systems.. Opt Lett.

[16] Popescu G, Ikeda T, Dasari RR, Feld MS (2006). Diffraction phase microscopy for quantifying cell structure and dynamics.. Opt Lett.

[17] Popescu G, Deflores LP, Vaughan JC, Badizadegan K, Iwai H (2004). Fourier phase microscopy for investigation of biological structures and dynamics.. Optics Letters.

[18] Joo C, Akkin T, Cense B, Park BH, de Boer JE (2005). Spectral-domain optical coherence phase microscopy for quantitative phase-contrast imaging.. Optics Letters.

[19] Rockward WS, Thomas AL, Zhao B, DiMarzio CA (2008). Quantitative phase measurements using optical quadrature microscopy.. Applied Optics.

[20] Chen BQ, Stamnes JJ (1998). Validity of diffraction tomography based on the first Born and the first Rytov approximations.. Applied Optics.

[21] Gbur G, Wolf E (2001). Relation between computed tomography and diffraction tomography.. J Opt Soc Am A Opt Image Sci Vis.

[22] Zysk AM, Reynolds JJ, Marks DL, Carney PS, Boppart SA (2003). Projected index computed tomography.. Opt Lett.

[23] Choi W, Fang-Yen C, Badizadegan K, Oh S, Lue N (2007). Tomographic phase microscopy.. Nat Methods.

[24] Charriere F, Marian A, Montfort F, Kuehn J, Colomb T (2006). Cell refractive index tomography by digital holographic microscopy.. Opt Lett.

[25] Charriere F, Pavillon N, Colomb T, Depeursinge C, Heger TJ (2006). Living specimen tomography by digital holographic microscopy: morphometry of testate amoeba.. Opt Express.

[26] Fiolka R, Wicker K, Heintzmann R, Stemmer A (2009). Simplified approach to diffraction tomography in optical microscopy.. Opt Express.

[27] Debailleul M, Georges V, Simon B, Morin R, Haeberle O (2009). High-resolution three-dimensional tomographic diffractive microscopy of transparent inorganic and biological samples.. Optics Letters.

[28] Maire G, Drsek F, Girard J, Giovannini H, Talneau A (2009). Experimental Demonstration of Quantitative Imaging beyond Abbe's Limit with Optical Diffraction Tomography.. Physical Review Letters 102.

[29] Wang Z, Millet L, Mir M, Ding H, Unarunotai S (2011). Spatial light interference microscopy (SLIM).. Opt Express.

[30] Mir M, Wang Z, Shen Z, Bednarz M, Bashir R (2011). Optical measurement of cycle-dependent cell growth.. Proc Natl Acad Sci U S A.

[31] Wang Z, Chun IS, Li XL, Ong ZY, Pop E (2010). Topography and refractometry of nanostructures using spatial light interference microscopy.. Optics Letters.

[32] Wang Z, Marks DL, Carney PS, Millet LJ, Gillette MU (2011). Spatial light interference tomography (SLIT).. Optics Express.

[33] Van Aert S, Van Dyck D, den Dekker AJ (2006). Resolution of coherent and incoherent imaging systems reconsidered – Classical criteria and a statistical alternative.. Optics Express.

[34] Sarder P, Nehorai A (2006). Deconvolution methods for 3-D fluorescence microscopy images.. Ieee Signal Processing Magazine.

[35] Wallace W, Schaefer LH, Swedlow JR (2001). A workingperson's guide to deconvolution in light microscopy.. Biotechniques 31: 1076–1078, 1080, 1082 passim.

[36] Cotte Y, Toy MF, Pavillon N, Depeursinge C (2010). Microscopy image resolution improvement by deconvolution of complex fields.. Optics Express.

[37] Haldar JP, Wang Z, Popescu G, Liang ZP (2011). Deconvolved Spatial Light Interference Microscopy for Live Cell Imaging.. Ieee Transactions on Biomedical Engineering.

[38] Babacan SD, Wang Z, Do M, Popescu G (2011). Cell imaging beyond the diffraction limit using sparse deconvolution spatial light interference microscopy.. Biomed Opt Express.

[39] Donachie WD (1993). The Cell-Cycle of Escherichia-Coli.. Annual Review of Microbiology.

[40] Shih YL, Le T, Rothfield L (2003). Division site selection in Escherichia coli involves dynamic redistribution of Min proteins within coiled structures that extend between the two cell poles.. Proceedings of the National Academy of Sciences of the United States of America.

[41] Donachie WD (2001). Co-ordinate regulation of the Escherichia coli cell cycle or The cloud of unknowing.. Molecular Microbiology.

[42] Raskin DM, de Boer PAJ (1999). Rapid pole-to-pole oscillation of a protein required for directing division to the middle of Escherichia coli.. Proceedings of the National Academy of Sciences of the United States of America.

[43] Sun Q, Margolin W (1998). FtsZ dynamics during the division cycle of live Escherichia coli cells.. Journal of Bacteriology.

[44] Ghosh AS, Young KD (2005). Helical disposition of proteins and lipopolysaccharide in the outer membrane of Escherichia coli.. Journal of Bacteriology.

[45] Wang Z, Popescu G (2010). Quantitative phase imaging with broadband fields.. Applied Physics Letters 96: -.

[46] Wang Z, Millet L, Chan V, Ding HF, Gillette MU (2011). Label-free intracellular transport measured by spatial light interference microscopy.. Journal of Biomedical Optics 16.

[47] Wang R, Wang Z, Millet L, Gillette MU, Levine AJ (2011). Dispersion-relation phase spectroscopy of intracellular transport.. Optics Express.

[48] Mir M, Tangella K, Popescu G (2011). Blood testing at the single cel level using quantitative phase and amplitude microscopy.. Biomed Opt Exp.

[49] Wang Z, Balla A, Tangella K, Popescu G (2011). Tissue refractive index as marker of disease.. J Biomed Opt.

[50] Sridharan S, Mir M, Popescu G (2011). Simultaneous optical measurement of cell motility and growth.. Biomed Opt Exp.

[51] Donoho D (2004). For most large underdetermined systems of linear equations the minimal L1-norm near solution approximates the sparsest solution.. Technical Report.

[52] Szameit A, Shechtman Y, Osherovich E, Bullkich E, Sidorenko P (2012). Sparsity-based single-shot subwavelength coherent diffractive imaging.. Nat Mater.

[53] Shechtman Y, Eldar YC, Szameit A, Segev M (2011). Sparsity based sub-wavelength imaging with partially incoherent light via quadratic compressed sensing.. Opt Express.

[54] Babacan SD, Molina R, Katsaggelos AK (2010). Sparse Bayesian Image Restoration. IEEE International Conference on Image Processing (ICP 2010).. Hong Kong.

[55] Nikolova M, Ng MK (2005). Analysis of half-quadratic minimization methods for signal and image recovery.. Siam Journal on Scientific Computing.

[56] Allain M, Idier J, Goussard Y (2006). On global and local convergence of half-quadratic algorithms.. Ieee Transactions on Image Processing.

[57] Ding H, Wang Z, Nguyen F, Boppart SA, Popescu G (2008). Fourier Transform Light Scattering of Inhomogeneous and Dynamic Structures.. PRL 101.

